# The Fate of Lipid Biosignatures in a Mars-Analogue Sulfur Stream

**DOI:** 10.1038/s41598-018-25752-7

**Published:** 2018-05-15

**Authors:** Jonathan Tan, James M. T. Lewis, Mark A. Sephton

**Affiliations:** 1 Impacts and Astromaterials Research Centre, Department of Earth Science and Engineering, Imperial College London, London, SW7 2AZ UK; 2 Present Address: NASA Goddard Space Flight Center, Greenbelt, MD 20771 USA

## Abstract

Past life on Mars will have generated organic remains that may be preserved in present day Mars rocks. The most recent period in the history of Mars that retained widespread surface waters was the late Noachian and early Hesperian and thus possessed the potential to sustain the most evolved and widely distributed martian life. Guidance for investigating late Noachian and early Hesperian rocks is provided by studies of analogous acidic and sulfur-rich environments on Earth. Here we report organic responses for an acid stream containing acidophilic organisms whose post-mortem remains are entombed in iron sulphates and iron oxides. We find that, if life was present in the Hesperian, martian organic records will comprise microbial lipids. Lipids are a potential sizeable reservoir of fossil carbon on Mars, and can be used to distinguish between different domains of life. Concentrations of lipids, and particularly alkanoic or “fatty” acids, are highest in goethite layers that reflect high water-to-rock ratios and thus a greater potential for habitability. Goethite can dehydrate to hematite, which is widespread on Mars. Mars missions should seek to detect fatty acids or their diagenetic products in the oxides and hydroxides of iron associated with sulphur-rich environments.

## Introduction

The martian surface environment has changed through time and the rock record has preserved evidence of a series of mineralogically-distinct Eras^1–4^. Early Noachian Mars displayed wet and more Earth-like conditions that led to the production of widespread hydrated phyllosilicates^1,2,5–7^. The diminishment of the martian atmosphere during the late Noachian^8,9^ in conjunction with increasing volcanic activity, led to acidic, sulfur-rich conditions and the regional deposition of sulfate salts in aqueous conditions that continued throughout the Hesperian^6,10–12^. There then followed an environment characterized by oxidising and dry conditions dominated by iron oxides, which has existed for a majority of Mars history, from 3.0 Ga up to and including the present day^1^.

It has been suggested that the preservation of life’s organic compounds may be difficult in Fe^3+^ mineral-containing sedimentary deposits owing to a predisposition for oxidation reactions^13,14^, yet contrary evidence has been provided by molecular organic extracts of ferruginous sediments from recent^15^ and ancient^16^ deposits from natural acid streams on Earth (e.g. Rio Tinto), while another study found that up to 21% of organic carbon in sediment is bound to Fe-bearing phases^17^. Lipid preservation studies have already been conducted in other Fe-rich environments such as iron hot springs, and suggest that the encrustation of cells by iron oxide minerals can enhance the initial preservation of lipids by rendering them unavailable for enzymatic degradation by heterotrophs^18,19^.

**Figure 1:**
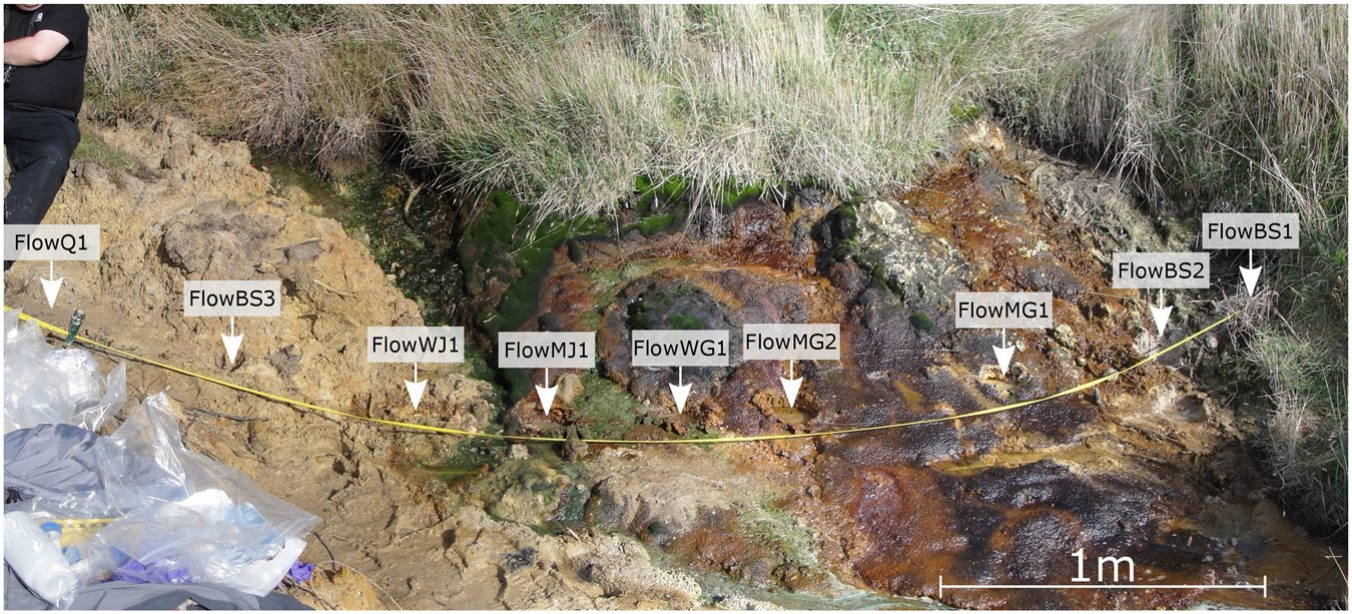
Overview of a ferric sulfate-rich stream in Dorset, Southern England. The study area is approximately 4 m across, and the locations of each of the sampled cores are shown. Ferric iron and sulfates are derived from the oxidation of pyrite in surrounding sedimentary rocks, giving the water a pH of 3.5.

**Figure 2:**
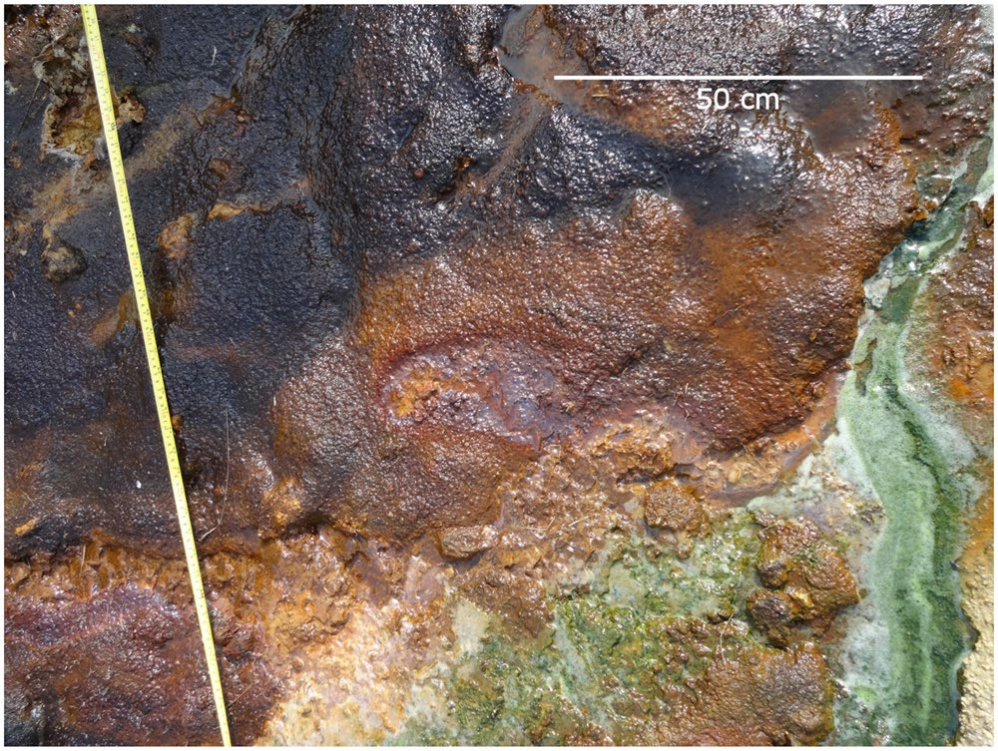
A closeup of the mineralogy present in the stream. Jarosite (yellow) is precipitated and where water-to-rock ratios are high and/or the pH increases, jarosite can be converted to goethite (reddish-brown). The stream hosts a distinct microbial community, including acidophilic algae (green) and microbial mats (purple).

Acidic streams on Earth are proposed as appropriate mineralogical environments to study as analogues of Mars^20–23^. We collected samples from an acidic stream (pH 3.5) located in St. Oswald’s Bay, Dorset (Figs 1 and 2), in which ferric sulfate-rich streamwaters derived from the aqueous oxidation of pyrite in surrounding sedimentary rocks have led to the precipitation of jarosite, in a process that is analogous to the provenance of some martian jarosite^24^. Goethite is also observed that may have been formed together with jarosite^24^, or produced by the transformation of jarosite, in line with established understanding of reactions in iron-rich sediments^25,26^.

Acidophilic algae, purple mat-forming sulfur bacteria and wood fragments from the adjacent bankside represent the primary organic inputs into the acid stream system (Fig. 2). Bacteria and algae contain a range of microbial lipids, including fatty acids, that can be used to consider the possible fate of similar lipids from any microbes on Mars while the wood fragments contain oxidized and degraded organic structures, such as phenols and polycyclic aromatic hydrocarbons (PAHs), which bear some superficial chemical similarities to meteoritic organic matter^27^ and can represent abiotic organic matter delivered to the martian surface as meteorite infall. The acid stream organic assemblage and its mineralogical context provides us with an opportunity to study the early stage diagenesis of lipids in an acidic, Fe-rich environment that acts as a mineralogical and geochemical analogue of conditions suggested by deposits detected on Mars.

We prepared gram-sized samples by grinding and homogenization in a sterilized, porcelain pestle and mortar. Mineral contents were assessed using X-ray diffraction (XRD) (Table 1). Lipid contents were extracted using sonication and centrifugation and an initial monophase water:methanol:chloroform solvent mixture followed by the addition of more water and chloroform to produce a two phase solution with any lipids isolated in the chloroform layer^28^. The chloroform extract was reduced by evaporation and derivatized with a boron trifluoride and methanol mixture to form fatty acid methyl esters (FAME). This lipid fraction was then analyzed by gas chromatography-mass spectrometry (GC-MS).

**Table 1:** Samples from flowing and dry acidic, ferric sulfate-rich streams. XRD data from^47^.

Sample	Code	Mineralogy (wt %) or Biomass type	cm from W bank
*Flowing stream*			
Acidophilic algae	FlowAL	Algae	
Bank sediment (W)	FlowBS1a	Grass	0
	FlowBS1b	Q:69,G:0,J:1,I:21,K:8,M:1	
	FlowBS2a	Wood	30
	FlowBS2b	Q:64,G:0,J:8,I:12,K:15,M:1	
Matt over goethite	FlowMG1a	Q:64,G:26,J:0,I:0,K:0,M:0	85
	FlowMG1b	Q:23,G:72,J:5,I:0,K:0,M:0	
	FlowMG1c	Q:89,G:0,J:5,I:0,K:4,M:2	
	FlowMG2a	Q:40,G:59,J:0,I:0,K:1,M:0	150
	FlowMG2b	Q:53,G:47,J:0,I:0,K:0,M:0	
	FlowMG2c	Q:89,G:0,J:7,I:0,K:3,M:1	
Wood over goethite	FlowWG1a	Wood	190
	FlowWG1b	Q:55,G:45,J:0,I:0,K:0,M:0	
	FlowWG1c	Q:80,G:0,J:0,I:13,K:7,M:0	
	FlowWG1d	Q:96,G:0,J:7,I:0,K:0,M:4	
Matt over jarosite	FlowMJ1a	Q:56,G:19,J:2,I:18,K:4,M:1	225
	FlowMJ1b	Q:99,G:0,J:0,I:0,K:0,M:1	
	FlowMJ1c	Q:73,G:0,J:26,I:0,K:0,M:1	
Wood over jarosite	FlowWJ1a	Wood	260
	FlowWJ1b	Q:65,G:0,J:27,I:0,K:8,M:0	
	FlowWJ1c	Q:63,G:0,J:28,I:0,K:9,M:0	
Bank sediment (E)	FlowBS3a	Q:81,G:0,J:2,I:9,K:7,M:1	325
	FlowBS3b	Wood	
	FlowBS3c	Q:79,G:0,J:1,I:10,K:9,M:1	
	FlowBS3d	Q:58,G:0,J:41,I:0,K:0,M:1	
	FlowBS3e	Q:72,G:0,J:1,I:15,K:11,M:1	
Quartz sand	FlowQ1	Q:87,G:0,J:1,I:7,K:4,M:1	380
*Dry stream*	DryMJ1a	Q:87,G:18,J:6,I:25,K:11,M:0	
	DryMJ1b	Q:43,G:38,J:19,I:0,K:0,M:0	
	DryMJ1c	Q:23,G:72,J:5,I:0,K:0,M:0	
	DryMJ1d	Q:32,G:56,J:12,I:0,K:0,M:0	

**Figure 3:**
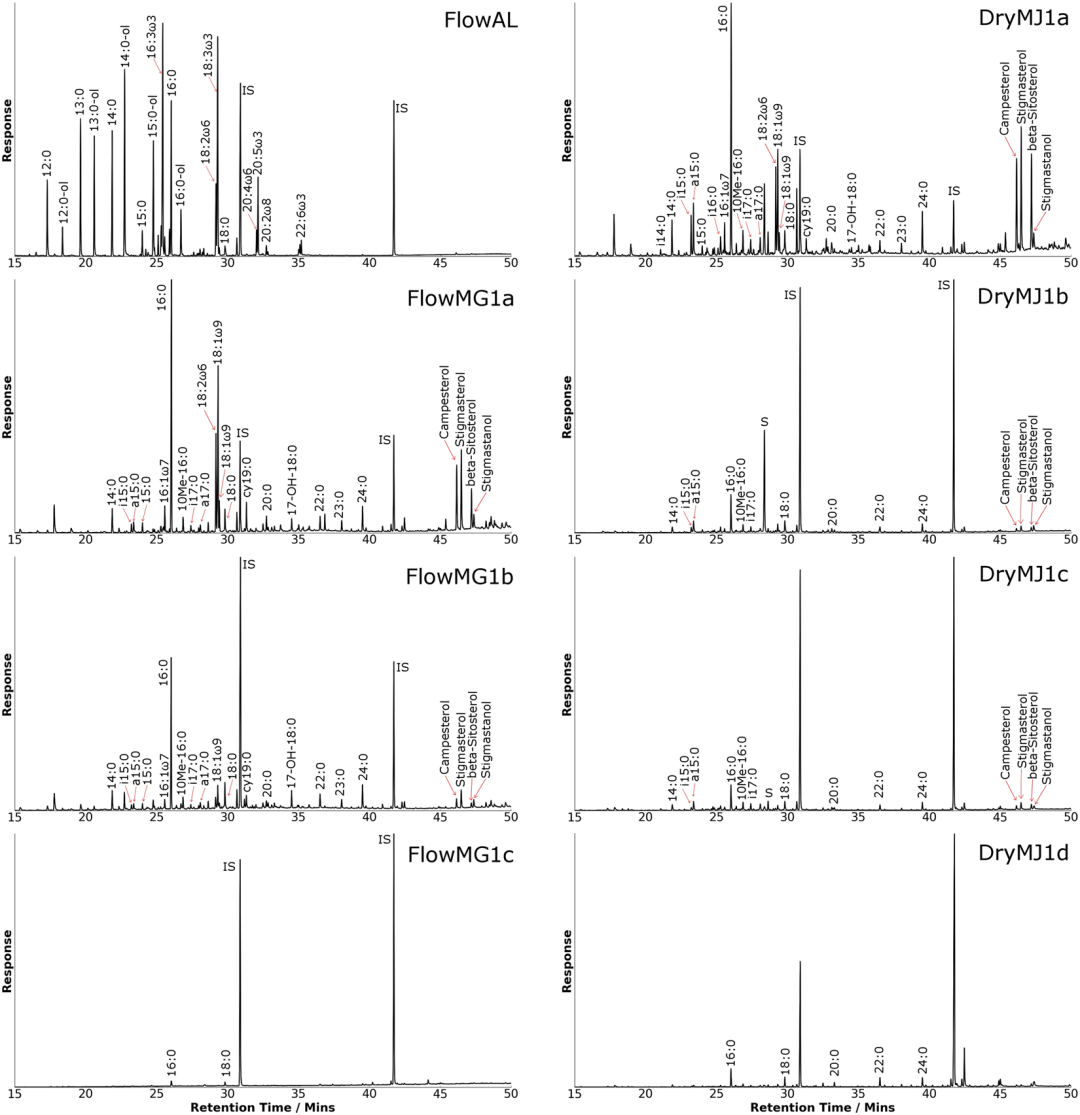
Total ion current chromatograms displaying lipids obtained from solvent extraction of the flowing and dry acidic, ferric sulfate-rich streams, as well as from the algae.

Our acid stream organic matter provided a glimpse into how the general markers of life may be preserved in martian environments of similar chemistries (Fig. 3). In the acid stream biomass, saturated straight-chain fatty acids were by far the most predominant indicators of life, consistent with their recognized high abundance in bacterial membrane phospholipids^29–31^. The acid stream fatty acids exhibit clearly recognizable even-over-odd (EOP) predominances in carbon-chain length and the C_16_ and C_18_ saturated fatty acids were particularly ubiquitous, consistent with the knowledge that these two species of acid are the most common saturated fatty acids in organisms^29,31^.

In addition to general indicators of life, some lipids from the acid stream samples were specific to certain types of organisms or biosynthetic pathways. The presence of terminally branched saturated fatty acids, i15:0, a15:0, and a17:0, and mid-chain branched 10Me16:0 detected in FlowMG1a are biomarkers for sulfate-reducing bacteria^32–36^. Cyclopropyl fatty acids appeared only in the microbial mat samples, consistent with their association with anaerobic bacteria^33,34,37^. ω-7 monounsaturated fatty acids (with a double bond 7 carbon atoms from the methyl end), such as 11-octadecenoic acid are formed by anaerobic-desaturase pathways, and are thus markers for anaerobic biosynthesis^29,35,37,38^, though aerobes are capable of using this pathway as well^34^. In contrast, ω-9 monounsaturated fatty acids such as 9-octadecenoic are produced by an oxygen-mediated biosynthetic pathway^38^, and are characteristic of cyanobacterial lipid profiles^35^. If life ever arose on Mars, it is unlikely that its evolutionary development would have extended to land plants; hence the fossil derivatives of ω-7 and cyclopropyl fatty acids would be available as use as microbial markers of anaerobic and aerobic metabolism^34^. Polyunsaturated fatty acids such as 20:4ω6, 20:5ω3, and 22:6ω3 are considered markers of microalgae^39^.

Some acid stream compounds are specific to higher plants with the polycyclic terpene phytosteroids such as campesterol, stigmasterol and β-sitosterol detected in the microbial mat samples FlowMG1a, FlowMG2a, FlowMJ1a and DryMJ1a. Goethite samples FlowMG1b, FlowMG2b, FlowWG1b and DryMJ1c also exhibited limited abundances of these compounds. Higher plants never evolved on Mars, but the fate of their steroid remains can suggest how more Mars-relevant polycyclic terpenes, such as bacterial hopanoids, may behave during fossilization under Mars-like conditions. Long-chain alcohols were observed in most of the microbial mat samples, and are derived from wax esters that are not found in purple sulfur bacteria^40,41^, and are instead markers of plant surface lipids^42^, which are likely to be of limited relevance to Mars.

**Figure 4:**
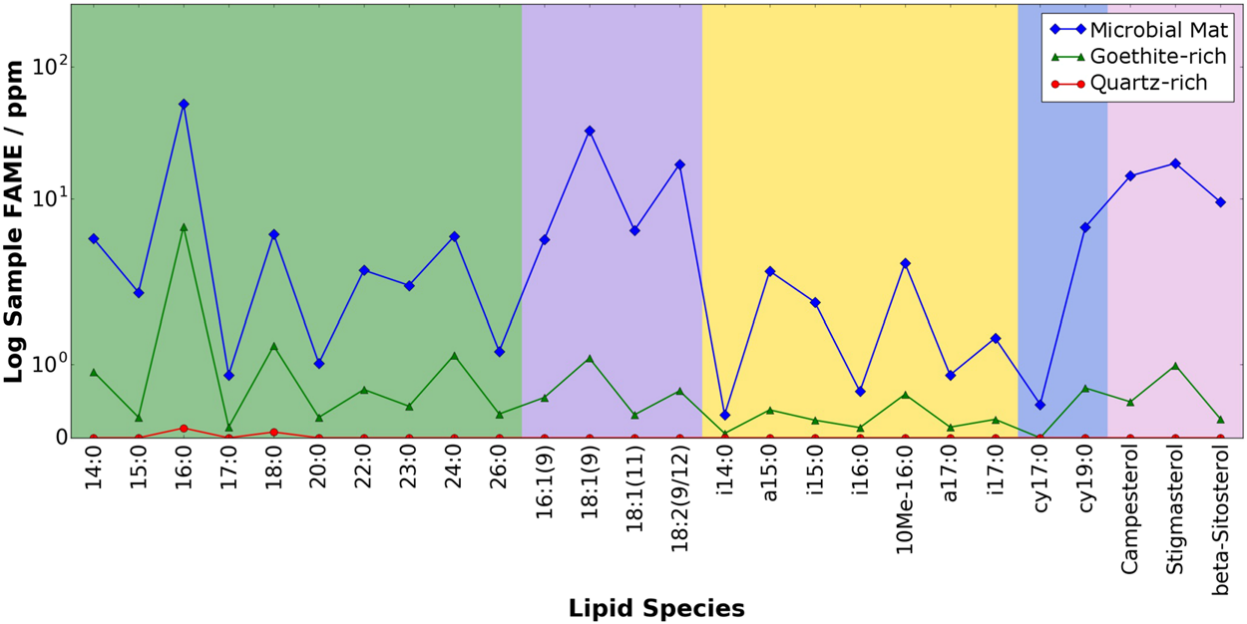
Lipid abundances (ppm) from three different mineralogies. The core displayed, with increasing depth, a microbial mat, a goethite-rich sediment from the flowing acidic ferric sulfate-rich stream (FlowMG1a – FlowMG1c) and underlying quartz sands and clays (comprised of minor kaolinite and microcline). Lipid classes are indicated by different colored backgrounds and categorised as follows: green, saturated fatty acids; purple, unsaturated fatty acids; yellow, branched fatty acids; blue, cyclopropyl fatty acids; pink, hydroxy-fatty acids; brown, phytosterols. Lipid abundances are much greater within the goethite-rich section compared to the clay-rich section of the core. FAME = Fatty Acid Methyl Esters. Note the logarithmic scale.

Diagenesis is controlled by microbial action or chemical reactions catalyzed by mineral surfaces, and leads to the reduction in abundance and diversity of all lipid compounds with increasing depth^43^. Almost all acid stream cores in our study display a decrease in fatty acid abundances with depth over centimeter scales (Table 2, Fig. 4). The rate of decrease in fatty acid abundances during diagenesis appears related to the mineralogical host. Goethite layers appear to preserve fatty acids relatively well including the retention of biogenic signatures such as EOP in the saturated fatty acids and the retention of unsaturated fatty acids to some extent. More complex organic compounds such as the polycyclic terpenes preserve less well in our acid stream environment, irrespective of the mineralogical host. Exceptions are DryMJ1c, which experienced repeated instances of stream flow, and the flowing stream bank sediment (FlowBS3d and FlowBS3e) where a wood layer occurred at the middle of the core. Because the acidic stream data can only be confidently extrapolated to the initial stages of diagenesis, it is important to understand how these markers would appear in a fossilized state in order to identify biomarkers in ancient martian sulfate environments. Microbial degradation also causes changes to the chemistry of the lipids, including processes such as defunctionalization. Most of the long-chain fatty acids containing oxygen-functional groups will undergo dehydration and decarboxylation resulting in saturated hydrocarbons^30^. Thermal diagenesis over long timescales causes the decarboxylation of the saturated fatty acids and their EOP pattern will be converted to a corresponding odd-over-even predominance (OEP) in saturated hydrocarbons^44–46^. For our acid stream samples, the characteristic C_19_ cyclopropyl fatty acid would convert to C_18_ mid-chain mono-methyl alkanes and these compounds have been recognized previously in cyanobacterial mats^40^.

**Table 2:** Fatty acid concentrations (ppm) flowing and dry acidic, ferric sulfate-rich streams.

Sample	Code	C_16_ FA	C_18_ FA	Total FA	cm from W bank
*Flowing stream*					
Acidophilic algae	FlowAL	176.0	17.9	1530.8	
Bank sediment (W)	FlowBS1a	158.3	104.3	679.3	0
	FlowBS1b	35.5	21.6	93.7	
	FlowBS2a	42.2	16.4	575.9	30
	FlowBS2b	6.8	4.5	36.6	
Matt over goethite	FlowMG1a	173.3	38.7	572.3	85
	FlowMG1b	27.7	8.2	58.9	
	FlowMG1c	2.0	1.4	3.3	
	FlowMG2a	272.7	25.2	455.1	150
	FlowMG2b	11.3	4.3	42.6	
	FlowMG2c	1.9	1.2	3.1	
Wood over goethite	FlowWG1a	60.4	41.6	196.4	190
	FlowWG1b	31.2	6.0	62.4	
	FlowWG1c	5.0	1.8	9.1	
	FlowWG1d	3.1	1.4	7.1	
Matt over jarosite	FlowMJ1a	62.1	16.4	226.8	225
	FlowMJ1b	3.0	2.0	5.0	
	FlowMJ1c	2.3	1.4	18.7	
Wood over jarosite	FlowWJ1a	13.3	4.2	76.9	260
	FlowWJ1b	4.9	2.2	13.3	
	FlowWJ1c	2.1	1.2	4.6	
Bank sediment (E)	FlowBS3a	7.0	6.9	22.9	325
	FlowBS3b	5.7	3.6	103.6	
	FlowBS3c	2.9	1.9	75.8	
	FlowBS3d	6.1	4.2	11.2	
	FlowBS3e	3.6	3.2	25.9	
Quartz sand	FlowQ1	4.1	1.7	5.8	380
*Dry stream*	DryMJ1a	91.1	22.2	338.6	
	DryMJ1b	4.7	2.7	14.4	
	DryMJ1c	9.6	5.7	31.1	
	DryMJ1d	6.0	3.3	28.2

There also appears to be a mineralogical influence on the initial preservation of the acid stream organic biosignatures. Concentrations of lipids are highest in goethite layers which represent environments of heightened aqueous activity conducive to habitation, and hence predisposed to receiving heightened microbial fossil inputs. The dependence of lipid records on conditions that promote habitation is further demonstrated by the paucity of lipids in the quartz sand and clay layers immediately beneath the goethite, which are not directly exposed to lipid inputs, as well as the jarosite-rich layers adjacent to the stream, which indicate environments of lower water activity and pH, and thus less favourable conditions for the growth of organisms. Moreover, goethite has been suggested recently as a high priority mineral for sampling because of its ability to originate from the transformation of jarosite^25^ and host organic matter, yet avoid the negative effects of oxygen generation and organic combustion during thermal extraction^47,48^. The lipid extract data now reveal that goethite-rich sedimentary materials contain relatively high concentrations of biosignatures irrespective of the analytical extraction methods employed.

A possible concern is that the higher abundance and diversity of lipids found in the goethite and jarosite layers can be attributed to the close association between these sublayers and the extant microbial mat, as opposed to the preservation potential of iron oxides. Contributions from extant biomass can be identified by the presence of intact polar membrane lipids, such as phospholipids or glycolipids, that degrade rapidly in 9–12 hours after cell death^49^. Analysis of the lipid fraction of the goethite layers revealed no phospholipids or glycolipids to a detection limit of 0.05 ppm and there were, therefore, no significant contributions from living biomass to the lipids detected in the goethite layer. All lipids extracted from the goethite layers must therefore be fossil molecules and not inadvertent contributions from the overlying microbial mat.

Terrestrial microbes contain 1 to 10% fatty acids^50^ suggesting that a past martian biosphere could have contained substantial amounts of fatty acids. The goethite-containing layers in samples from the acid streams contained between 31.05 to 58.94 ppm of total fatty acids respectively. Non-volcanic Hesperian sediment occupies approximately 16.099 × 10^6^ km^2^ of the martian surface^51^. The uppermost centimetre of Hesperian terrain represents a volume of 1.610 × 10^11^ m^3^ of material and a mass of 4.857 × 10^14^ kg. If the fatty acid concentrations of the Hesperian sediments on Mars are equivalent to those in the Hesperian analogue acidic sulfur stream then the most shallow centimetre layer would potentially contain up to 1.50 × 10^10^ kg (0.015 Gt) to 2.86 × 10^10^ kg (0.029 Gt) of fatty acids that survive initial diagenesis.

It must be noted that our calculations contain a number of assumptions. Mars may not have supported an equivalent microbial biomass as exhibited at the acid stream, and many of the postulated fatty acids on Mars could have been transformed to alkanes by enzymatic^52^ or diagenetic^30^ processes or destroyed by the ionizing effects of radiation^53^ or the oxidising effects of perchlorates that are abundant on the martian surface^54^. It is also pertinent to note that Mars would not have had available a similarly efficient aerobic metabolism as found on the present day Earth^55^ and the lack of martian plate tectonics would have led to different burial processes and associated thermal effects. Differences between diagenesis on Earth and Mars may partly explain why the Sample Analysis at Mars instrument on the Mars Science Laboratory Curiosity rover was able to detect only chlorinated benzenes in surface rocks^48^. Nevertheless, this data provides conceptual guidance for present and future Mars missions, and suggests focusing the search for evidence of fatty acid monomers and/or their diagenetic products in goethite deposits that are mineralogical signposts of aqueous, and thus more habitable conditions.

## Methods

### Samples

Samples were collected from a field site located in St. Oswald’s Bay, Dorset, United Kingdom, where a small stream was flowing from slumped Wealden Beds that are rich in pyrite (Figs 1 and 2). The water was acidic (pH 3.5) and jarosite was observed in many of the sedimentary layers surrounding the stream. There were substantial lateral variations across the study site. Where water-to-rock ratios and/or pH were higher the jarosite was closely associated with goethite. Biology was evident in the form of a purple microbial mat that partially covered the goethite layer, and acidophilic algae were observed as green particulates within the stream. To the east of St Oswald’s Bay, at Stair Hole, a locality of similar facies was observed but was found to be mostly dried out except for a small flow of pH 5 water. Stream samples were extracted as cylindrical cores of 10 cm diameter and 10–15 cm depth using a trowel. The samples were packaged in aluminum foil and freeze died. Individual biological and mineral layers were separated using a solvent-cleaned saw (washed with methanol and DCM) to ensure that there was no contamination in the core, and subsequently powdered with a pestle and mortar as described in the literature^47^ to allow for X-ray diffraction and lipid analysis.

### X-Ray diffraction (XRD)

The samples were analysed using a Philips PW 1830 between 2.5 and 90 °2Ө with a step size of 0.02 and two seconds per step. The X’Pert HighScore program was used to analyse the diffraction patterns and perform Rietveld refinements. Quantification of crystalline phases was possible to an accuracy of 1–3 wt%.

### Lipid Extraction

Between 0.5 g and 2 g of each sample were loaded into centrifuge tubes and 10 µl of internal standard added (1 mg ml^−1^ of 5-β cholanic acid and 0.5 mg ml^−1^ of p-terphenyl). A monophase solvent was prepared (4:10:5 ratio of water:methanol:chloroform; Bligh and Dyer, 1959) and 4 ml added to the sample. The mixture was subjected to sonication (5 min) and centrifugation (3 min, 1800 rpm). The supernatant was transferred to stoppered test tubes and the process repeated three times. Chloroform and water were then added to the test tubes, shaken and left to settle, resulting in immiscible layers of clear water over lipid-rich chloroform. The chloroform was pipetted into round-bottomed flasks, and the process repeated three times, before the combined extracts were reduced by rotary evaporation (40 °C). Dichloromethane (DCM) and anhydrous sodium sulfate were added to the extract to remove any remaining chloroform and water. The resulting solution was pipetted into 4 ml vials for derivatization.

### Lipid Derivatization

The DCM extracts were dried down under nitrogen gas. 1 ml of boron trifluoride-methanol (BF_3_-methanol) solution was added to the vials and heated at 80 °C for 40 min. After cooling, 1 ml of water and 1 ml of hexane were added to the vials forming immiscible layers of hexane over water, with the hexane layer containing the methyl esters of any carboxylic acids present. The hexane layer was collected using a pipette, transferred to a short alumina column and eluted with 1:1 DCM:hexane. The eluate was evaporated under nitrogen gas prior to analysis.

### Gas Chromatography-Mass Spectrometry

GC-MS analysis utilised an Agilent Technologies 7890 GC coupled to a 5973 MS. Injection (1 μl) was splitless with helium carrier gas at a constant flow rate of 1.1 mL min^−1^. Separation was performed on a J&W DB-5MS UI column (approximately 30 m in length, 0.25 mm internal diameter, and a film thickness of 0.25 μm). The GC oven temperature was held at 40 °C for 2 minutes and then ramped at 5 °C min^−1^ to 310 °C, where it was held for 9 minutes. Mass spectra were acquired in the scan range (45–550 amu). Peak identification was based on retention time and mass spectra comparisons with authenticated standards and by reference to published reports. In particular, we used standards for stearic acid (18:0) and oleic acid (18:1(9)); 18:1(11) was assumed to be the additional peak with identical mass spectra to oleic acid (18:1(9)) owing to its status as the other of the two most common monounsaturated fatty acids in bacterial cell membranes^30^. The peak assigned as cyclopropyl fatty acid was identified owing to its unique mass spectra; the assignment is restricted to the class and molecular weight with no positional detail relied upon for our interpretations.
